# Numerical Assessment of Damage Parameters for a Hard Interface Model

**DOI:** 10.3390/ma15155370

**Published:** 2022-08-04

**Authors:** Maria Letizia Raffa, Raffaella Rizzoni, Frédéric Lebon

**Affiliations:** 1 Laboratoire QUARTZ EA 7393, ISAE-Supméca, 93400 Saint-Ouen-sur-Seine, France; 2 Department of Engineering, University of Ferrara, 44122 Ferrara, Italy; 3 Aix Marseille Université, CNRS, Centrale Marseille, LMA, 13453 Marseille, France

**Keywords:** imperfect interface, adhesive, micro-cracking, analytical modelling, identification

## Abstract

Adhesive interfaces are suitable modelling tools to describe very thin elastic layers and the related occurring phenomena (such as damage, viscosity, friction, etc.), without using a volumetric description, which is often computationally prohibitive in a large-scale numerical simulation. A major drawback of these kinds of models is the identification of free parameters, because of the smallness of a direct observation scale. This paper proposes a numerical assessment of two model parameters, a damage energy threshold and a damage viscosity, of a hard interface model previously formulated by authors. The proposed assessment protocol uses macroscopic experimental data, available in the literature, on structural adhesives under standard characterization tests. The numerical results obtained give insights into the physical interpretation of these parameters.

## 1. Introduction

Adhesive interface modelling has been an expanding branch of solid mechanics research since the early 1940s. Goland and Reissner [1] were pioneers in modelling a thin adhesive as a weak interface and they were the first to define the spring-type interface, by assuming that the adherents were bonded by a continuous distribution of springs. They preconized that the thinness would involve a uniform distribution of the stresses field in the adhesive, and some years later, Gilibert and Rigolot [2] found a rational justification of this fact by means of the asymptotic expansion method, assuming that the thickness and the elastic properties of the adhesive had the same order of smallness ε.

During the eighties and nineties, the relaxation of the perfect interface approximation, i.e., continuity at the interfaces in displacements and stresses fields, was largely investigated, aiming to apply these theories to composite materials with coated fibres [3,4] or particles [5,6], or to the decohesion and nucleation problems in cohesive zones [7,8,9]. Many efforts were made to model damage [10,11], and other related physics such as friction [12,13] and viscosity [14], in adhesive interfaces. All these models have the advantage to allow a macroscopic description of a thin elastic adhesive and the occurring phenomena, without using a volumetric description, which is often computationally prohibitive in a large-scale numerical simulation.

A major drawback of these kinds of models is the identification of free parameters. Often, the identification protocols need direct experimental observations and this is not a trivial issue in very thin layers because of the small scale of the phenomena to be investigated. A fallback solution is to build on macroscale experimental tests on adhesive specimens and adhesive joint specimens and try to correlate microscopic model parameters to a macroscopic response [15].

The hard imperfect interface model with damage formulated by authors in [16] has been chosen to apply an estimation procedure of damage parameters. In particular, this interface model is suitable to describe structural adhesives as stiff as substrates [17]. In this case, the mechanical behaviour of the adhesive cannot be accurately described via a classic spring-like interface model (i.e., continuity in stresses field and discontinuity of displacements field [1]), but a hard condition considering also a jump in the stresses field is more indicated, as demonstrated by [17].

The imperfect interface model that is adopted for this numerical study has two free parameters, which is a great advantage of this kind of phenomenological models. These parameters represent respectively a damage viscosity (η) and a damage energy threshold (ω) and they are related to the damage evolution law, as detailed in what follows. Note that the model sensitivity on these parameters has been already investigated by authors in [16], thus it is not the subject of this work.

This paper proposes a first attempt at a numerical assessment of the damage parameters η and ω. The proposed estimation procedure is based on experimental data available in the literature, which concerns macroscopic characterization tests on both bulk and joint structural adhesives commonly used in industry.

## 2. Materials and Methods

### 2.1. Overview of the Hard Imperfect Interface Model for Micro-Cracked Adhesive Joints

In this section, a brief overview of the imperfect interface model is proposed. For an extensive description of the formulation, one refers to [16]. The model consists of a law of hard imperfect contact coupled with a damage evolution law. It is able to describe the mechanical behaviour of structural adhesives with micro-cracking damage. The transmission conditions reported below, prescribe jumps in the stresses $[[\sigma \;e_{3}]]$ and displacements [[u]] fields across an interface of outward normal unit vector $e_{3}$ between two adherents, thus describing the asymptotic behaviour of a very thin deformable adhesive made of a general anisotropic linear elastic material:

(1) $$[[u]]=\;\varepsilon \left({\left(K^{33}\right)}^{-1}\left(\langle \langle \sigma \;e_{3}\rangle \rangle -K^{\alpha 3}\langle \langle u_{,\alpha }\rangle \rangle \right)-\langle \langle u_{,3}\rangle \rangle \right),$$

(2) $$\begin{array}{cc} {}[[\sigma \;e_{3}]]= & \varepsilon ({\left(-K^{\beta \alpha }\langle \langle u_{,\beta }\rangle \rangle -K^{3\alpha }{\left(K^{33}\right)}^{-1}\left(\langle \langle \sigma \;e_{3}\rangle \rangle -K^{\beta 3}\langle \langle u_{,\beta }\rangle \rangle \right)\right)}_{,\alpha }\\ & -\langle \langle \sigma_{,3}\;e_{3}\rangle \rangle ), \end{array}$$

where ε is the thickness of the adhesive, the symbols [[(·)]] and 〈〈(·)〉〉 are taken to denote the jump and the average of the quantity (·) across the interface separating the two adherents, respectively; Greek indexes (α,β=1,2) are related to the in-plane ($x_{1},x_{2}$) quantities; commas denote first derivatives and the summation convention is used. Matrices $K^{ij},i,j=1,2,3$ are related to the elasticity coefficients $B_{ijkl}$ of the adhesive layer (from the constitutive equation in linear elasticity: $\sigma_{ij}=B_{ijkl}\;e_{kl}$). If the adhesive is modelled as isotropic, with Young’s modulus $\bar{E}$ and Poisson’s ratio $\bar{\nu },$ the matrices $K^{ij}$ read as:

(3) $$\begin{array}{ccc} K^{ii} & = & \frac{\bar{E}}{2(1+\bar{\nu })}\;\left(\frac{2(1-\bar{\nu })}{\left(1-2\bar{\nu }\right)}\;e_{i}⊗e_{i}+e_{j}⊗e_{j}+e_{k}⊗e_{k}\right),\;\;i\neq j\neq k, \end{array}$$

(4) $$\begin{array}{ccc} K^{ij} & = & \frac{\bar{E}}{2(1+\bar{\nu })}\;\left(e_{i}⊗e_{j}+\frac{2\bar{\nu }}{\left(1-2\bar{\nu }\right)}\;e_{j}⊗e_{i}\right),\;\;j\neq i. \end{array}$$

Engineering moduli $\bar{E}$ and $\bar{\nu }$ represent the effective mechanical properties of an isotropic microcracked material. Note that the assumption of a random distribution of microcracks is considered here, thus the resulting material remains isotropic. In the section below, three different micromechanical homogenization schemes are used to derive the elastic moduli of the microcracked adhesive material.

### 2.2. Micromechanical Homogenization Schemes for a Microcracked Adhesive

Drawing on micromechanical homogenization theory [18,19,20,21,22,23] within the framework of effective field schemes, the effective elastic moduli of the microcracked adhesive are derived. Micro-cracking damage is represented here by a microcracks density parameter. Particularly, a generalized crack density [24] is adopted, allowing to by-pass the geometrical definition of the cracks, which is possible only for circular and regular cracks [25], and as a matter of fact, extending the generality of the interface model to any regular and irregular cracks shape. It should also be noted that the generalized microcracks density can be measured *postmortem* by X-ray micro-tomography [26,27].

#### 2.2.1. Kachanov-Sevostianov Scheme

The Kachanov-Sevostianov (KS) scheme [21,28] is a stress-based homogenization approach based on the non-interacting microcracks approximation (NIA) [25,29] and on Eshelby’s theory [18]. In the 2D case, assuming the adhesive is an initial isotropic matrix embedding a random distribution of microcracks, the elastic potential in stresses (complementary energy density) of the effective medium yields the following structure for the effective Young’s modulus $\bar{E}$ and Poisson’s ratio $\bar{\nu }$:

(5) $$\begin{array}{ccc} \bar{E} & = & E_{0}\;{\left(1+2\;C_{ks}\;E_{0}\;\rho \right)}^{-1}, \end{array}$$

(6) $$\begin{array}{ccc} \bar{\nu } & = & \nu_{0}, \end{array}$$

where $E_{0}$ and $\nu_{0}$ are the moduli of the undamaged matrix or the initial moduli of the adhesive before the damage, ρ is the generalized microcracks density (i.e., damage parameter) and the constant $C_{ks}$ depends on the orientational distribution of defects. For a 2D random distribution of microcracks, $C_{ks}=\frac{\pi }{E_{0}}$ [21,22]. Note that Equation (6) is an assumption valid for all considered schemes.

#### 2.2.2. Welemane-Goidescu Scheme

The Welemeane-Goidescu (WG) scheme [30,31,32,33] is a strain-based homogenization approach based on the dilute limit approximation (for further details about dilute limit and NIA, one refers to [28]). Under the hypothesis of a 2D random distribution of microcracks embedded in an isotropic matrix, a linearized expression of the effective Young’s modulus is found:

(7) $$\bar{E}=E_{0}\;\left(1-2\;\;C_{wg}\;E_{0}\;\rho \right),$$

with $C_{wg}=C_{ks}=\frac{\pi }{E_{0}}$ [31].

#### 2.2.3. Pan-Weng Scheme

Pan-Weng (PW) scheme [23] draws on Mori-Tanaka’s theory [34] and it is a stress-based homogenization approach. In analogy with the previously cited schemes, the 2D case of a randomly distributed microcracks family is considered. Accordingly, the effective Young’s modulus reads as:

(8) $$\bar{E}=E_{0}\;{\left(1-C_{pw}\;\rho \right)}^{-1},$$

where $C_{pw}=\frac{16}{3}\left(\nu_{0}^{2}-1\right)$ [23].

Note that by including Equations (5), (7) or (8) in the expression of the interface stiffness tensor (Equation (4)), a different dependency of the imperfect interface law on the crack density ρ, is obtained. Particularly, it has been previously established by authors that the KS scheme allows us to describe imperfect interfaces with ductile damaging behaviour, while the WG scheme is suitable to describe interfaces with brittle damaging behavior [16].

### 2.3. A Description for the Micro-Cracking Damage Evolution

In this section, a possible description of the evolving behaviour of the cracks density ρ is given by drawing on further works by authors [11,16]. Damage in the adhesive joint is assumed to be caused by a microcracks accumulation and damage parameter ρ is assumed to strictly increase in time. The evolution of the cracks density in the bulk adhesive of thickness ε (i.e., cohesive damage) can thus be described by the following first-order ODE proposed in [11]:

(9) $$\eta^{\varepsilon }\dot{\rho }={\left\{\omega^{\varepsilon }-\frac{1}{2}\;B_{,\rho }^{\varepsilon }\left(\rho \right)\left(e\left(u^{\varepsilon }\right):e\left(u^{\varepsilon }\right)\right)\right\}}_{+},$$

where ${\left\{\cdot \right\}}_{+}$ denotes the positive part of the function, $B_{,\rho }^{\varepsilon }\left(\rho \right)$ indicates the component-wise derivative of the effective stiffness tensor $B^{\varepsilon }\left(\rho \right)$ of the micro-cracked adhesive with respect to the generalized cracks density ρ, and e(u) is the strain tensor under the small perturbation hypothesis.

By applying asymptotic expansions theory to Equation (9) under the assumption of *hard interface* [17], the expression of the evolution of cracks density in the interface (i.e., adhesive damage) is obtained, as in [16]:

(10) $$\eta \dot{\rho }={\left\{\omega -\frac{1}{2}\;K_{,\rho }\left(\rho \right)\left(\begin{array}{c} \langle \langle u_{,1}\rangle \rangle \\ \langle \langle u_{,2}\rangle \rangle \\ {}[[u]]+\varepsilon \langle \langle u_{,3}\rangle \rangle \end{array}\right).\;\left(\begin{array}{c} \langle \langle u_{,1}\rangle \rangle \\ \langle \langle u_{,2}\rangle \rangle \\ {}[[u]]+\varepsilon \langle \langle u_{,3}\rangle \rangle \end{array}\right)\right\}}_{+},$$

with initial condition $\rho \left(0\right)=\rho_{0}$.

In Equation (10), u is the displacement field, symbols [[(·)]] and 〈〈(·)〉〉 are taken to denote the jump and the average of the quantity (·) across the interface and $K_{,\rho }\left(\rho \right)$ indicates the component-wise derivative of the stiffness tensor

(11) $$K\left(\rho \right)=\left(\begin{array}{ccc} \varepsilon K^{11} & \varepsilon K^{21} & K^{31}\\ \varepsilon K^{12} & \varepsilon K^{22} & K^{32}\\ K^{13} & K^{23} & \frac{1}{\varepsilon }K^{33} \end{array}\right)$$

with respect to the cracks density ρ.

### 2.4. Numerical Assessment Procedure

The adopted hard imperfect interface model (Equations (1) and (2)) integrating the damage evolution (Equation (10)) has two model parameters: η and ω (respectively $\eta^{\varepsilon }$ and $\omega^{\varepsilon }$ in the bulk adhesive). In detail, η (or $\eta^{\varepsilon }$) is a strictly positive constant parameter and represents a damage viscosity influencing the velocity of damage evolution; ω (or $\omega^{\varepsilon }$) is a strictly negative constant parameter and has the meaning of an energy threshold, after which damage evolution begins [16]. The goal of this work is to provide a first numerical assessment of these parameters.

**Figure 1:**
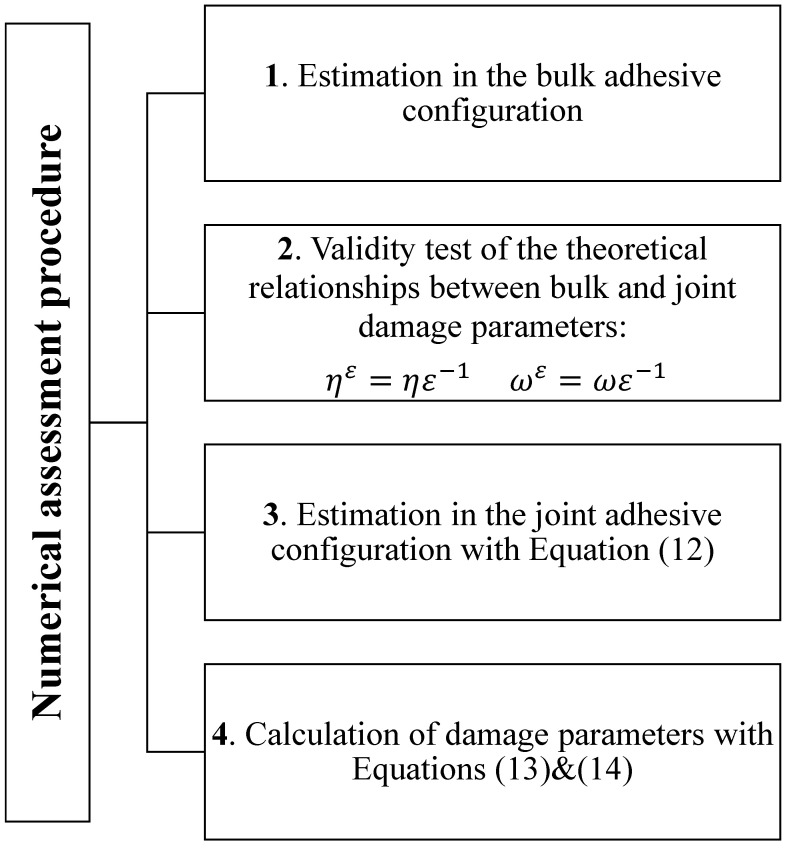
Flow chart detailing the numerical assessment procedure.

The proposed assessment protocol uses macroscopic experimental data on structural adhesives under standard characterization tests. To this aim, experimental data from Murakami and coworkers [35] concerning tensile and torsional tests on the epoxy-based structural adhesive XA7416 (3M Japan Ltd., Tokyo, Japan) and from Kosmann and coworkers [36] concerning torsional test on epoxy adhesive Henkel Hysol EA9695 AERO 0.05 NW (Henkel AG and Co., Düsseldorf, Germany), were chosen. Experimental data were extracted from [35,36] by using the free online software *WebPlotDigitizer* [37]. Numerical simulations were carried out by numerically solving the differential problem Equations (1) and (2) with Equations (10) and (11), using the commercial software *Mathematica* [38]. The estimation procedure is schematized in the flow chart in Figure 1.

**Figure 2:**
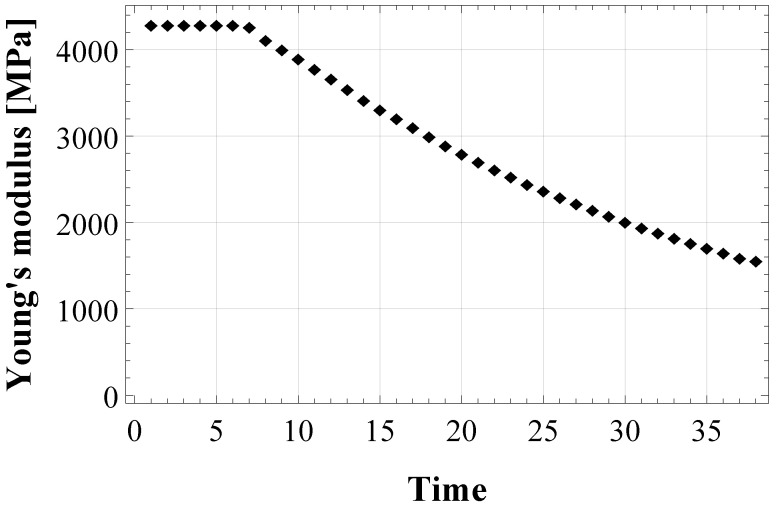
Evolution of the experimental Young’s modulus of the epoxy-based adhesive XA7416, calculated from tensile test data by [35].

First, an estimation process in the bulk configuration is performed. To this aim, experimental results of a tensile test on a bulk adhesive specimen (Figure 7 in [35]) have been used to calculate the evolution of the experimental Young’s modulus in time, shown in Figure 2.

Figure 2 shows that at the beginning of the test the Young’s modulus is equal to its initially undamaged value $E_{0}$, then micro-cracking begins, which decreases the modulus. Three different numerical models of $E(\dot{\rho })$ have been obtained by integrating Equation (9) in the three schemes for damaged material: Equation (5) (for KS model), Equation (7) (for WG model) and Equation (8) (for PW model). Then, the numerical models were fitted to the experimental data by the least-square minimization method, to derive the damage parameters in the bulk adhesive configuration, $\eta^{\varepsilon }$ and $\omega^{\varepsilon }$ in Equation (9).

Then, the theoretical damage parameters in the joint (interface) configuration η and ω have been calculated from relationships $\eta^{\varepsilon }=\eta \;\varepsilon^{-1}$ and $\omega^{\varepsilon }=\omega \;\varepsilon^{-1}$ and they have been used to calculate numerical curves and compared to experimental data. Since the theoretical parameters did not prove reliable for simulating experimental behaviour, a further step must be taken to identify damage parameters in the joint configuration.

An estimation process in the adhesive joint configuration was performed to identify damage parameters in the interface (η and ω). To this aim, shear strain-stress data of pure torsional tests on cylindrical butt-joint specimens were extracted from [35,36]. In this case, the analytical closed-form solution for the proposed hard interface model with damage (Equations (1) and (2) with (10)) has been formulated in [39] and represents the shear strain-stress (γ−τ) response of the adhesive joint:

(12) $$\gamma =\left\{\begin{array}{cc} a\tau , & 0\leq \tau \leq \tau_{0}\\ a\tau +b\tau {\left(\tau -\tau_{0}\right)}^{2}\left(\tau +2\tau_{0}\right), & \tau >\tau_{0} \end{array}\right.$$

where $\tau_{0}$ is the damage initiation shear stress. Equation (12) was fitted to the experimental shear strain-stress curves (cf. Figure 10 by [35] and Figure 8 by [36]) to derive model parameters a,b and $\tau_{0}$, then the damage parameters, η and ω, were estimated by using the following expressions:

(13) $$\begin{array}{ccc} \omega & = & -\frac{\tau_{0}^{2}\varepsilon \;C_{G}}{2\;G_{0}}, \end{array}$$

(14) $$\begin{array}{ccc} \eta & = & \frac{\varepsilon \;C_{G}^{2}}{6\;b\;\dot{\tau }\;G_{0}^{2}}. \end{array}$$

where $G_{0}$ is the initially undamaged shear modulus of the adhesive joint and $C_{G}$ is the microstructural parameter depending on the adopted damaged material model. It can be equal to $C_{ks},C_{wg}$ or $C_{pw}$ (see Equations (5), (7) and (8)).

For the numerical simulations of the cylindrical butt-joint specimens under torsion, the parameters related to the experimental configurations of the two different adhesive materials were extracted from [35,36] and reported in Table 1.

## 3. Results

### 3.1. Numerical Assessment in the Bulk Configuration

**Figure 3:**
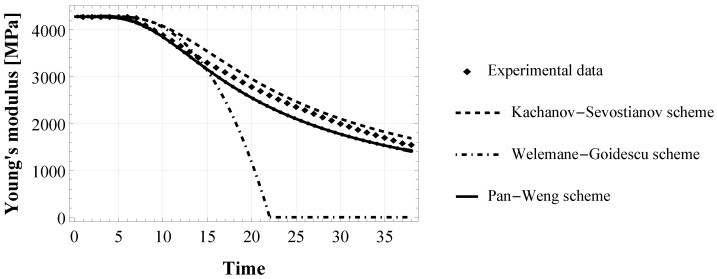
Evolution of the Young’s modulus of the epoxy-based adhesive XA7416: fitting of the proposed numerical model (with Kachanov-Sevostianov, Welemane-Goidescu and Pan-Weng scheme) to the experimental data extracted by authors from experiments by [35].

The behaviour of the apparent Young’s modulus of the epoxy-based adhesive XA7416 is represented in Figure 2. At the beginning of the tensile test (elastic domain), it is constant and equal to $E_{0}=4282$ MPa, then it degrades in time as an effect of the micro-cracks accumulation. This experimental finding was used to assess damage parameters in the bulk adhesive. Figure 3 shows the three numerical models obtained by implementing the evolution of the cracks density (Equation (9)) in the three schemes for damaged material, i.e., Equation (5), (7) and (8), respectively for KS, WG, and PW scheme.

Numerical results obtained with KS and PW model are quite similar and very close to the experimental behaviour, showing a gradual degradation of the Young’s modulus from its initial value $E_{0}$ until reaching 36% of $E_{0}$ at the end of the simulated tensile test. On the contrary, the WG model immediately after the onset of damage accumulation, corresponding at a strain of 0.4%, goes to zero abruptly. Note that time in this study is only indicative, so no units are necessary.

Damage parameters estimated in the bulk adhesive configuration are the same for all numerical models as reported in Table 2.

### 3.2. Numerical Assessment in the Joint Configuration

**Figure 4:**
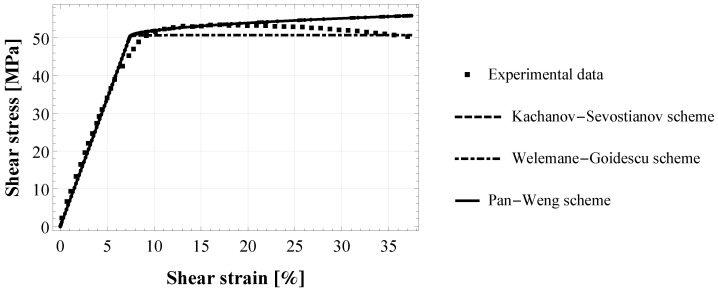
Torsional shear strain-stress (γ−τ) response of cylindrical butt-joint specimens of epoxy-based adhesive XA7416: fitting of the proposed numerical model (with Kachanov-Sevostianov, Welemane-Goidescu and Pan-Weng scheme) to the experimental data by [35].

Figure 4 shows the experimental behaviour under pure torsion of two S45C carbon-steel cylinders joined by an epoxy adhesive XA7416 [35] (see Table 1 for the characteristics of the experimental configuration). The elastic part of the experimental (γ−τ) curve has been used to estimate the initially undamaged shear modulus of the adhesive joint, which results in $G_{0}=672$ MPa. Moreover, in Figure 4 are represented the three best-fitted numerical curves for KS, WG and PW model, obtained from Equation (12) by using the same estimates for all three models, namely a=1.5829 × $10^{-3},\;b=1.0541$ × $10^{-6}$ and the damage initiation stress $\tau_{0}=50.25$ MPa. Then, the damage parameters ω and η were calculated from Equations (13) and (14), respectively, by using the proper microstructural parameter $C_{G}$, and reported in Table 2.

#### Influence of the Adhesive Type

**Figure 5:**
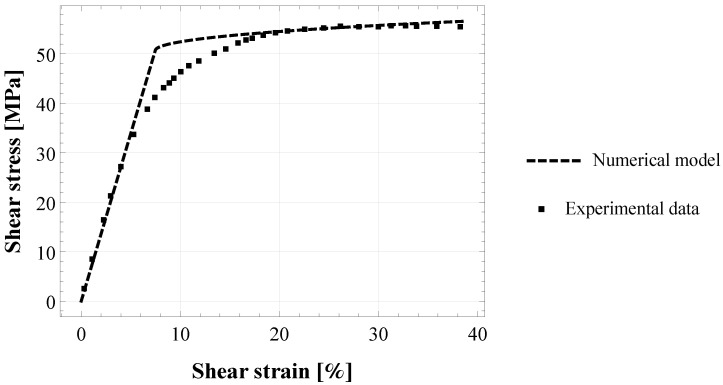
Torsional shear strain-stress (γ−τ) response of cylindrical butt-joint specimens of epoxy-based adhesive Henkel Hysol EA9695: fitting of the proposed numerical model (with Kachanov-Sevostianov scheme) to the experimental data by [36].

The experimental data by [36] were chosen to investigate the influence of the adhesive material type on the estimated parameters. Figure 5 shows the experimental behaviour under torsion of two steel cylinders joined by an epoxy adhesive Henkel Hysol EA9695 AERO 0.05 NW [36] (see Table 1 for the characteristics of the experimental configuration). The elastic part of the experimental (γ−τ) curve has been used to estimate the initially undamaged shear modulus of the adhesive joint, which results in $G_{0}=673$ MPa. Moreover, in Figure 5 is represented the best-fitted numerical curve for KS scheme, obtained from Equation (12) by using the following estimates: a=2.2224 × $10^{-3},\;b=9.9261$ × $10^{-7}$ and the damage initiation stress $\tau_{0}=50.77$ MPa. Then, the damage parameters ω and η were calculated from Equations (13) and (14), respectively, by using the proper microstructural parameter $C_{G}$ for the KS scheme, and the estimated parameters are reported in Table 2.

## 4. Discussion

The present work is a first attempt at coping with the identification of damage parameters for interface models with damage. One of the main questions that arises is whether these parameters represent an intrinsic property of the adhesive material, or they depend on the model adopted to describe the damage. For this reason, two numerical assessment procedures in both bulk and joint adhesive configuration are carried out by using three different homogenization schemes integrated into our hard interface model with damage evolution. Moreover, two different adhesive materials are studied.

Concerning the numerical assessment in the bulk configuration, the three damaged material models give the same damage threshold ω and damage viscosity η, as reported in Table 2. This result suggests that these parameters are related to the adhesive material nature as intrinsic properties, regardless of the model adopted. Moreover, the KS and PW model give very similar results, as shown in Figure 3, and are able to reproduce the asymptotic degradation of the experimental elastic modulus. This is related to the fact that both KS and PW micromechanical homogenization schemes are drawn on the approximation of non-interacting cracks. This assumption extends the accuracy of these schemes to high values of crack density (for ρ>60% as established in [28]). On the contrary, the WG scheme remains accurate to a lower density value than the first two models, and this is because it is based on the dilute limit assumption. These results are thus consistent with the theory [28,40].

As well-established in literature, the mechanical behaviour of the bulk adhesive can be very different from that of the adhesive joint, and this can explain why the damage parameters assessed in the joint configuration are different from that of the bulk adhesive configuration. Note that in [16] it is assumed that $\eta^{\varepsilon }$ and $\omega^{\varepsilon }$ are volumetric densities and thus inversely proportional to the non-dimensional interphase thickness ε: $\eta^{\varepsilon }=\eta \;\varepsilon^{-1}$ and $\omega^{\varepsilon }=\omega \;\varepsilon^{-1}$. Actually, the results obtained by numerical estimation disprove this hypothesis, as shown in Table 2.

As in the bulk configuration, also in the joint case, KS and PW give very similar results, the two curves are superposed as shown in Figure 4. It is worth highlighting that the numerical simulations of the pure torsional test on epoxy adhesive XA7416 are carried out in a force-controlled mode to be consistent with the experimental configuration, by imposing a shear stress rate $\dot{\tau }$. For this reason, numerical results, regardless of the considered homogenization scheme, cannot reproduce the experimental softening behaviour occurring for a shear strain γ>23%.

Concerning the numerical assessment in the joint configuration, the resulting damage parameters depend on the considered damage scheme, unlike what is found in the bulk configuration assessment. Particularly, damage parameters depend on the microstructural parameter $C_{G}$ (see Equations (13) and (14)). KS and WG schemes have the same microstructural parameter $C_{G}=C_{ks}=C_{wg}=\frac{\pi }{E_{0}}$ and this explains why the same damage parameters are found. Damage parameters estimated for the PW scheme are slightly different even if they have the same order of magnitude (see Table 2). Moreover, all the numerical curves, regardless of the scheme, are in good agreement with the experimental pattern. This result is consistent with previous numerical insights found in [39].

Figure 5 shows the comparison between the numerical model and the experimental data on cylindrical butt-joint with epoxy-based adhesive Henkel Hysol EA9695 by [36]. In this case, only the KS scheme was used in order to compare the resulting parameters with them estimated for the other adhesive in a similar joint configuration. It results that the estimated parameters η and ω are quite smaller than that obtained for the epoxy adhesive XA7416, as highlighted in Table 2. This fact can be due to the influence of the material’s elastic properties, although no precise information is available from [36] concerning bulk properties of adhesive Henkel Hysol EA9695. Another possible explanation is the influence of the experimental configuration. In fact, although the test configurations of Murakami [35] and Kosmann [36] are quite similar (steel cylindrical adhesively bonded butt-joint under torsion), some main differences still remain, such as the adhesive thickness and the loading rate. In detail, the film thickness in Kosmann’s experiments is thinner and the loading rate is higher than that in Murakami’s experiments (see Table 1). Additionally, the geometrical parameters of the joint specimens (not reported here) are also different. However, further investigations on different adhesive materials are necessary to derive a correlation between model damage parameters and the above properties. Moreover, it is crucial to have the same test configuration in order to provide reliable comparisons.

This study has some main limitations. First, the proposed estimation protocol is based on macroscopic experimental data, such as a tensile test on bulk adhesive specimens and a torsional test on bonded tubular butt-joints, even if the model parameters to be estimated are related to the material constitutive behaviour (microscale). However, this is a very usual choice in experimental mechanics because of the difficulty to carry out direct mensuration of damage at the interface level. Second, the considered damage evolution law is a unique function of mechanical loadings (see Equation (10)), nevertheless it is established that other physics, such as environmental conditions, can contribute to the damage evolution. However, the interface model formulated by [16] can be readily generalized in order to account for multi-physics couplings as done for example by [41]. Third, the viscoplasticity and viscoelasticity typical of structural adhesives were not considered in the proposed model. Multi-physics coupling and viscosity aspects could be the object of further works. Lastly, because of a lack of experimental data available in the literature, this study uses only two kinds of adhesive materials for the estimation of model parameters. As a perspective, more adhesives should be compared in order to establish reliable correlations between adhesive material properties and damage parameters η and ω.

## 5. Conclusions

This paper proposes a first attempt at a numerical assessment of the damage parameters of viscosity η and energy threshold ω of the imperfect interface model previously formulated by authors in [16]. The proposed estimation procedure draws on experimental data available in the literature, concerning macroscopic characterization tests on a structural adhesive commonly used in industry, in both bulk (tensile test) and joint (torsional test) configuration.

Several points arise from this preliminary numerical assessment and main are related to the physical meaning of model parameters η and ω:

- **They could represent an intrinsic material property** as the performed numerical estimation on the bulk configuration gives the same values of parameters regardless of the homogenization scheme used.
- **They could depend on the adhesive configuration** in agreement with the fact that the mechanical behaviour of an adhesive in bulk configuration is different from that of the same adhesive in joint form. In fact, the estimated parameters in the joint configuration are different from that found in the bulk one.
- In the joint configuration, parameters **could depend on the adopted homogenization scheme**.
- **They could depend on the adhesive material properties and on the test configuration**, however further investigation is needed to elucidate this point.
- The theoretical relationships between parameters in the bulk and in the joint configuration, assumed to be inversely proportional to the adhesive thickness ($\eta^{\varepsilon }=\eta \;\varepsilon^{-1}$ and $\omega^{\varepsilon }=\omega \;\varepsilon^{-1}$) [11,16,17] is not fulfilled.

Moreover, unanswered issues still remain, such as:

- the dependency of the model parameters on the considered microstructure (i.e., the shape of the porosity), particularly the influence of the microstructural parameter $C_{G}$ could be investigated;
- the dependency of the model parameters on the type of structural adhesive, to this aim several adhesive materials could be compared.
- In the above case, the possible dependency on the test configuration must be eliminated by using the same configuration for all different adhesives.

To corroborate these preliminary numerical insights and to answer the above-cited open questions, it will be crucial to expand this study by setting up a hybrid numerical/experimental protocol of identification. Within future research, several adhesive materials should be compared and *ad hoc* test configurations should be studied to investigate the correlation between parameters and physical properties.

## Figures and Tables

**Table 1:** Experimental parameters of the structural adhesives in joint configuration.

Parameter	Epoxy-Based Adhesive XA7416 [35]	Epoxy-Based Adhesive Henkel Hysol EA9695 [36]
Adhesive thickness ε [mm]	0.3	0.05
Loading rate $\dot{\tau }$ [MPa/s]	6.67 × $10^{-2}$	9.14 × $10^{-1}$
Shear modulus $G_{0}$ [MPa]	671.88	672.91

**Table 2:** Numerically estimated damage parameters.

Configuration/Homog. Scheme/Adhesive Type	Damage Threshold ω [MJ/${\mathrm{mm}}^{2}$]	Damage Viscosity η [MJ·s/${\mathrm{mm}}^{2}$]
Bulk/KS, WG and PW/XA7416	−0.3	230
Joint/KS and WG/XA7416	−3.54	62.19
Joint/PW/XA7416	−2.62	33.95
Joint/KS/EA9695	−0.60	0.80

## Data Availability

No new data were created or analyzed in this study. Data sharing is not applicable to this article.
